# Engineering bacteria for biogenic synthesis of chalcogenide nanomaterials

**DOI:** 10.1111/1751-7915.13320

**Published:** 2018-10-17

**Authors:** Prithiviraj Chellamuthu, Frances Tran, Kalinga Pavan T. Silva, Marko S. Chavez, Mohamed Y. El‐Naggar, James Q. Boedicker

**Affiliations:** ^1^ Department of Physics and Astronomy University of Southern California Los Angeles CA USA; ^2^ Department of Biological Sciences University of Southern California Los Angeles CA USA; ^3^ Department of Chemistry University of Southern California Los Angeles CA USA

## Abstract

Microbes naturally build nanoscale structures, including structures assembled from inorganic materials. Here, we combine the natural capabilities of microbes with engineered genetic control circuits to demonstrate the ability to control biological synthesis of chalcogenide nanomaterials in a heterologous host. We transferred reductase genes from both *Shewanella* sp. ANA‐3 and *Salmonella enterica* serovar Typhimurium into a heterologous host (*Escherichia coli)* and examined the mechanisms that regulate the properties of biogenic nanomaterials. Expression of arsenate reductase genes and thiosulfate reductase genes in *E. coli* resulted in the synthesis of arsenic sulfide nanomaterials. In addition to processing the starting materials via redox enzymes, cellular components also nucleated the formation of arsenic sulfide nanomaterials. The shape of the nanomaterial was influenced by the bacterial culture, with the synthetic *E. coli* strain producing nanospheres and conditioned media or cultures of wild‐type *Shewanella sp*. producing nanofibres. The diameter of these nanofibres also depended on the biological context of synthesis. These results demonstrate the potential for biogenic synthesis of nanomaterials with controlled properties by combining the natural capabilities of wild microbes with the tools from synthetic biology.

## Introduction

Biological synthesis of nanomaterials provides exciting new routes to synthesize materials for use in environmental remediation, automobiles, photovoltaics, aircrafts, medical imaging and medical implants (Thakkar *et al*., 2010; Hulkoti and Taranath, 2014). Biogenic synthesis of nanomaterials is a process of creating nanomaterials using biochemical reactions and biomolecules from bacteria, fungi, plants and viruses. Microbes found in nature are capable of building nanoscale materials, such as magnetosomes and silicates (Cai *et al*., 2007; Yan *et al*., 2012). Microbes have also evolved redox enzymes capable of processing many starting materials used in semiconductor and metallic nanostructure, such as manganese, iron, selenium, arsenate, uranium and chromium (Liu *et al*., 2002; Nealson *et al*., 2002; Yan *et al*., 2012). These capabilities suggest the possibility of large‐scale synthesis of nanomaterials by cells, similar to the large‐scale biogenic synthesis of organic compounds, including biofuels and drugs (Cai *et al*., 2007; Yan *et al*., 2012). Cellular construction of nanomaterials can also leverage the continued advancement of genetic control circuits, enabling the combination and fine‐tuned control of synthesis pathways to modulate nanomaterial properties.

Nanomaterials, such as cadmium sulfide, arsenic sulfide, gold nanoparticles, silver nanoparticles and zinc oxide have previously been synthesized using microorganisms (Narayanan and Sakthivel, 2010; Quester *et al*., 2013; Jacob *et al*., 2016; Plaza *et al*., 2016). These materials include both nanomaterials composed of single elements, such as nanoparticles synthesized from gold or silver (Chandran *et al*., 2006; Kumar *et al*., 2010; Bharali *et al*., 2013), as well as nanomaterials composed of multiple elements, such as cadmium sulfide, arsenic sulfide and zinc sulfide (Bai *et al*., 2006; Lee *et al*., 2007; Bakhshi and Hosseini, 2016). For instance, arsenic sulfide nanofibres with semiconductor properties were synthesized by wild‐type *Shewanella* species (Lee *et al*., 2007; Jiang *et al*., 2009; McFarlane *et al*., 2015). Gold, Silver nanoparticles and CdTe/CdS quantum dots have been patterned using a biological template (Naik *et al*., 2002; Chen *et al*., 2014).

A major challenge in biogenic synthesis of nanomaterials is the control of nanomaterials properties, including size, shape and composition. Biomolecules such as glycolipids and proteins can control the size and shape of the nanomaterials (Mao *et al*., 2003; Kumar *et al*., 2010; Narayanan *et al*., 2010; Singh *et al*., 2011; Dunleavy *et al*., 2016; Dahoumane *et al*., 2017). A better understanding of the biochemical pathways and biomolecules involved in controlling the nanomaterial properties would help us create a better biological system with tunable circuits to influence the nanomaterial properties. For instance, do we need live cells to process the precursors or can we simply add cell extracts or purified proteins or glycolipids to control the nanomaterial properties? Current work has shown that for some nanomaterials, purified proteins and biomolecules were sufficient, however in other cases, an active microbial cell was needed (Kumar *et al*., 2010; McFarlane *et al*., 2015; Dunleavy *et al*., 2016). The shape of the nanomaterials plays an important role in controlling the optical properties and the application of the nanomaterial; star‐shaped copper oxide nanoparticles had the highest catalytic activity of reduction of 4‐nitrophenol by NaBH_4,_ compared with rod and spherical‐shaped nanoparticles (Konar *et al*., 2016). Similarly, the optical properties of gold nanoparticles were determined by the shape of the nanoparticles (Nehl and Hafner, 2008). Another critical variable in controlling the optical properties of the nanomaterial property is its size. When dealing with materials in the nanoscale, quantum confinement effect plays an important role in controlling the optical properties (Lin *et al*., 2015; Bajorowicz *et al*., 2018). For instance, when the size of the nanomaterial increases, there is a corresponding decrease in bandgap energy. Researchers observed an increase in size of the nanomaterials and a corresponding shift in photoluminescence emission of spherical CdTe quantum dots towards higher wavelength (Red‐shift) by increasing the incubation time with *E. coli* cells (Bao *et al*., 2010). Biological routes of synthesis offer new strategies for controlling nanoparticles properties.

In this work, we take advantage of the ability of microbes to change the oxidation state of metals and metalloids. Microbes have evolved both specific and non‐specific enzymes to transfer electrons to metals such as iron and manganese as well as metalloids such as selenium and arsenic. These redox reactions are either part of the respiratory chain or detoxification pathways. Here, we transfer redox pathways from two strains of bacteria, arsenate reductase genes from *Shewanella* sp. ANA‐3 and thiosulfate reductase gene from *Salmonella enterica* serovar Typhimurium, into a heterologous host to synthesize arsenic sulfide nanomaterials. Arsenic and thiosulfate reductase genes were cloned into plasmids and expressed under the control of inducible promoters (Bang *et al*., 2000a,b). Using this synthetic system, we investigated how the properties of synthesized nanomaterials depend on biological activity, finding that both redox activity and nucleating factors produced by the cells control the size and shape of the resultant nanomaterials.

## Results

### Engineering *E. coli* to reduce arsenate and thiosulfate

To create a synthetic microbial system capable of producing arsenic sulfide nanostructures, genes for arsenate and thiosulfate reduction were introduced via plasmids (Table 1) into a heterologous host, *E. coli*. *Escherichia coli* was chosen as the host organism because it is genetically well characterized with developed tools for synthetic biology applications and has successfully been used as a host strain for expression of *Shewanella* pathways (Malasarn *et al*., 2008; Ang *et al*., 2013; Jensen *et al*., 2016; Schuergers *et al*., 2017).

**Table 1:** List of plasmids used in this study

Plasmid	Backbone	Key genes	Source
pSB74	pTrc99A	Thiosulfate reductase *(phsABC)* induced by IPTG, AmpR	Addgene
pZE arsDABC	pZE (Reed *et al*., 2012)	Arsenate reductase *(arsDABC)* induced by IPTG, SmR (Spectinomycin)	This Study
pBAD arrAB	pTS1b (Shis and Bennett, 2013)	Arsenate reductase*(arrAB)* induced by arabinose, ChlR (Chloramphenicol)	This Study

For arsenate reduction, *arsDABC* (4.2 kb) and *arrAB* (3.2 kb) from *Shewanella* sp. ANA‐3 were assembled into two plasmids. The *arrAB* is an arsenic operon giving *Shewanella* sp. ANA‐3 the capability to respire arsenate As(V) as a terminal electron acceptor and couple it to growth. The *arsDABC* operon is a separate arsenate detoxification pathway from the same strain (Saltikov and Newman, 2003; Saltikov *et al*., 2003; Malasarn *et al*., 2008). Both pathways reduce arsenate As(V) to arsenite As(III) – necessary for the formation of arsenic sulfide nanomaterial.

For thiosulfate reduction, thiosulfate reductase gene (*phs*ABC)(Bang *et al*., 2000a,b) from *Salmonella enterica* Serovar Typhimurium was obtained from Addgene. Previously, researchers used this plasmid in an engineered system for bioremediation applications (Bang *et al*., 2000a,b). Plasmid maps (Fig. S1) and primers used (Table S1) are available in the supporting information.

**Figure 1:**
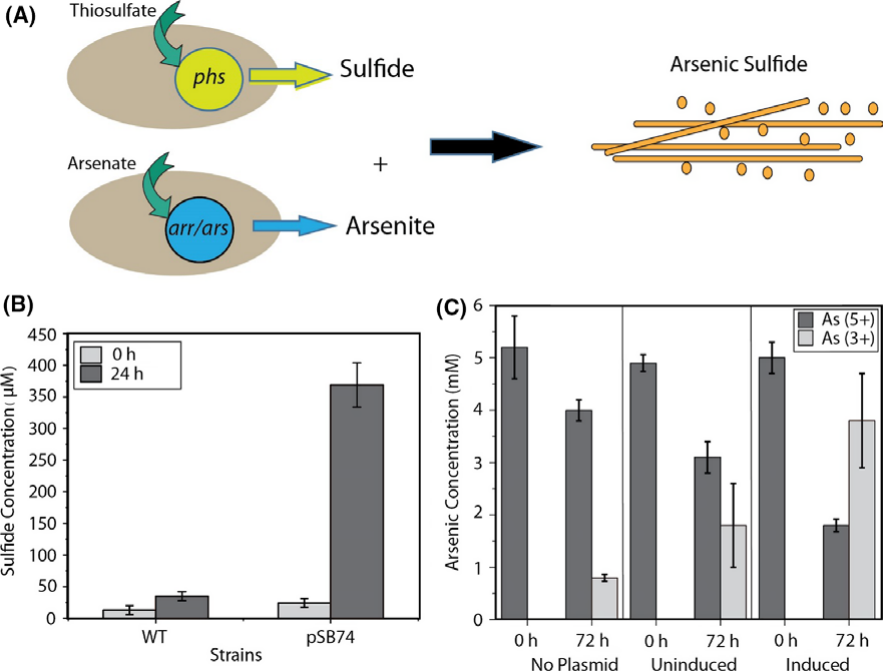
Reduction of thiosulfate and arsenate by engineered *E. coli*. A. Plasmids containing pathways for the reduction of arsenate (*arr/ars*) and thiosulfate (*phs*) were inserted into *E. coli* host cells, enabling the formation of arsenic sulfide nanomaterials. B. *E. coli* expressing the thiosulfate reductase produced high concentrations of sulfide. C. Arsenate reductase from *Shewanella sp*. ANA‐3 in *E. coli* increased the rate of arsenite production as compared with negative controls in uninduced cells or cells without the plasmid.

Figure 1A shows a schematic of engineered *E. coli* strains involved in thiosulfate reduction to sulfide and arsenate reduction to arsenite, two substrates necessary for arsenic sulfide production. The strains were co‐cultured for experiments involving the biogenic synthesis of arsenic sulfide nanomaterials.

In control experiments, *E. coli* without the plasmids were tested for the ability to reduce arsenate and thiosulfate. As shown in Fig. 1B, after 24 h, *E. coli* with plasmid pSB74 reduced 2.5 mM thiosulfate to 350 μM sulfide, 10‐fold more than no plasmid control. Similarly, arsenate reduction capabilities of *E. coli* strains with plasmids pZE arsDABC and pTSlb arrAB were quantified, as shown in Fig. 1C. When induced, strains carrying the arsenate reductase plasmids were able to reduce 5 mM arsenate to 3.8 mM arsenite at 72 h. Uninduced cultures generated 1.6 mM arsenite after 72 h. Background levels of arsenate reduction in cells without the plasmids produced 0.8 mM arsenite. A schematic of arsenate reduction in *E. coli* with cellular machinery involved in arsenate reduction and transportation of arsenate in and out of the cell is presented in Fig. S2. Sequence analogy of the proteins involved in arsenate reduction and transportation between *E. coli* and ANA‐3 is presented in Table S2.

### Arsenate as terminal electron acceptor in engineered *E. coli*

Arsenate has a thermodynamically favourable redox potential for an electron acceptor (E_0_: +560 mV, compare this with oxygen at +836 mV). In arsenate respiring bacteria, the organisms can derive energy for growth by reducing arsenate to arsenite using respiratory arsenate reductase enzymes such as ArrAB (Saltikov and Newman, 2003). Upon reduction, arsenite is exported out of the cell to limit toxicity. ArrAB is a heterodimer protein with molybdenum and ironsulfur cluster involved in arsenate reduction process (Malasarn *et al*., 2008).

We tested if *E. coli* cells could use arsenate as a terminal electron acceptor for growth. Elsewhere in the paper cells grown under anaerobic conditions used fumarate as an electron acceptor. To test for growth with arsenate as the sole electron receptor, *E. coli* expressing *arrAB* and *arsDABC* genes from ANA‐3 were added to minimal media with 500 μM of arsenate with no fumarate. There was no change in biomass represented by change in absorbance (OD_600_) and cell count (CFU ml^−1^) (data not shown). This indicates that engineered *E. coli* with the cloned genes *arsDABC* and *arrAB* could not facilitate arsenate respiratory capabilities, with an associated gain in energy and biomass.

### Synthesizing arsenic sulfide nanomaterials with engineered *E. coli*

**Figure 2:**
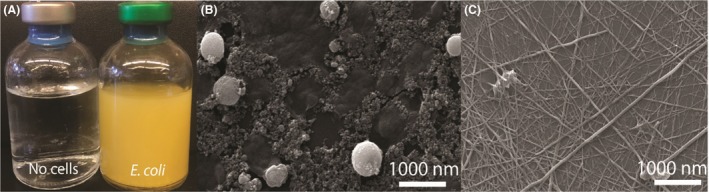
Biogenic production of arsenic sulfide nanomaterials. A. Bottles containing media without cells, and engineered *E. coli* expressing thiosulfate and arsenate reductases form yellow coloured arsenic sulfide precipitate. Scanning electron micrograph images of nanomaterials produced by engineered *E. coli* (B) and wild‐type *Shewanella sp*. ANA‐3 (C). Both samples were collected at 72 h.

We tested the ability of engineered *E. coli* cells with inducible thiosulfate reductase and arsenate reductase to synthesize arsenic sulfide nanomaterials. After induction of the reductase genes, arsenate and thiosulfate were added to the culture. *E. coli* cells expressing arsenate reductase genes formed arsenic sulfide precipitate after 24 h. Arsenite reacts with sulfide to form arsenic sulfide nanomaterial as show in Fig. 2A. Although sulfide alone has been shown to reduce As(V) to As(III), in control experiments shown in Fig. S3, this low level of arsenate reduction was not sufficient to generate any precipitate. Additional controls shown in Fig. S4 show that the dense, yellow precipitate is not due to *E. coli* growth. We also verified that cells containing either arsDABC or arrAB were able to synthesize spherical arsenic sulfide nanomaterials, see Fig. S5.

We then tested the effect of nanomaterial formation on cell viability. Arsenic sulfide formation had a deleterious effect on cell viability. In our experiments, within 24 h of initiation of arsenic sulfide precipitation, *E. coli* cell viability dropped to < 10 colony forming units per ml (CFU ml^−1^) as shown in Fig. S6. In controls without arsenic sulfide precipitates, the cell viability did not drop to zero CFU ml^−1^, but exhibited natural attenuation in viability as one would expect in a culture running out of nutrients.

The precipitate morphology was assessed using scanning electron microscopy. The engineered *E. coli* cultures formed spherical arsenic sulfide structures with an average diameter of 381 ± 246 nm (Fig. 2B), which was surprising given that arsenate and thiosulfate reduction by *Shewanella* sp*. *ANA‐3 is known to synthesize nanofibres of arsenic sulfide, as shown in Fig. 2C and previously reported (McFarlane *et al*., 2015). The shape of the material was influenced by the microorganism expressing the reductase genes. Energy‐dispersive X‐ray (EDX) spectroscopy was used to characterize the elemental composition of the precipitate from both *E. coli* and ANA‐3 cultures. The precipitates had signature characteristic peaks (Fig. S7) corresponding to arsenic and sulfur, confirming the yellow precipitate for both host strains was arsenic sulfide. Table 2 summarizes the nanomaterials formed for the conditions and strains described above.

**Table 2:** Characteristics of biogenic nanomaterials formed through cellular reduction of arsentate and thiosulfate

Cell type	Reactants	Precipitation	Nanomaterial	Size (nm)
*Shewanella* ANA‐3	Arsenate, thiosulfate	Yes	Fibre	46 ± 14
*E. coli* with pZE arsDABC and pBAD arrAB and *E. coli* with pSB74	Arsenate, thiosulfate	Yes	Spheres	411 ± 125

X‐ray diffraction characterization confirmed the presence of diffraction peaks corresponding to multiple phases of arsenic sulfide nanomaterials (Fig. S8), as reported in earlier work (Ledbetter *et al*., 2007; Lee *et al*., 2007; Rodriguez‐Freire *et al*., 2016). The diffrations patterns revealed overlapping crystalline phases that were produced by both *E. coli* and ANA‐3, such as orpiment (As_2_S_3_), realgar (AsS) and alacranite (As_8_S_9_). Previous reports have observed that the arsenic sulfide material crystallinity improves moderately over a period of time (Lee *et al*., 2007; McFarlane *et al*., 2015).

### Cellular components were needed to induce formation of arsenic sulfide nanomaterials

**Figure 3:**
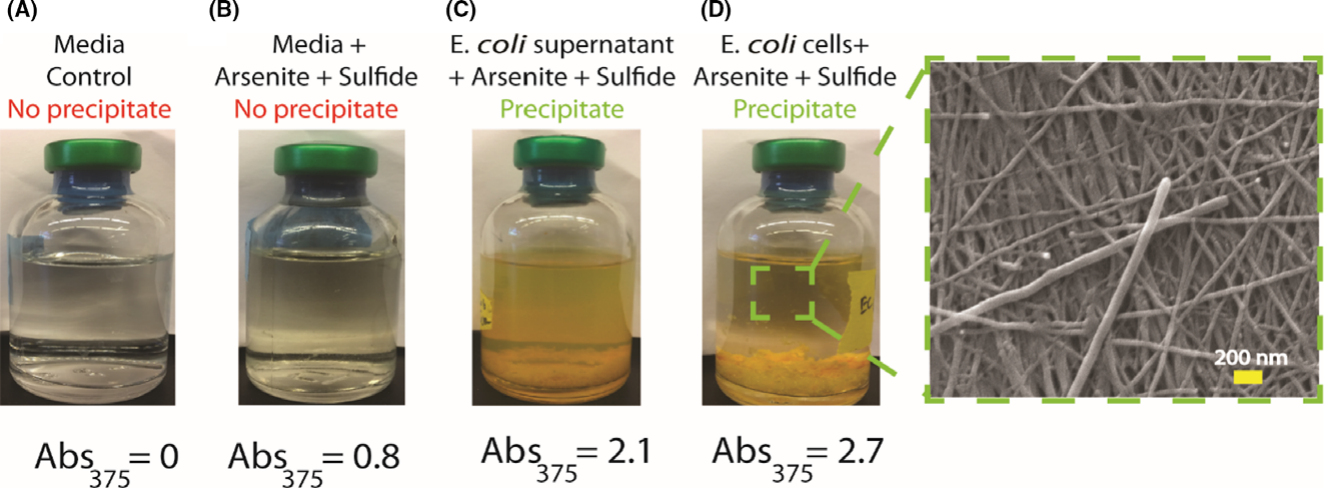
Cellular components were needed to precipitate arsenic sulfide nanomaterials. A. Control bottle of media with no arsenite and sulfide formed no precipitate. B. In the absence of cellular components, bottles with media, arsenite and sulfide formed no precipitate. Absorbtion of 375 nm light indicates trace amounts of arsenic sulfide material in solution. C, D. Bottles with arsenite and sulfide containing either cell‐free supernatant or *E. coli* cells produced solutions with high absorbance at 375 nm and large amounts of yellow precipitate, characteristic of arsenic sulfide materials. SEM images revealed arsenic sulfide nanofibres were produced when arsenite and sulfide were added to *E. coli* cultures.

Control experiments were run to determine what roles the cells played in nanomaterial synthesis other than the reduction of the starting arsenate and thiosulfate. To identify additional roles of bacterial cells in nanomaterial formation, reduced substrates, 5 mM arsenite and 10 mM sulfide were added to cultures of cells and media without cells. Bottles containing minimal media did not form precipitate (Fig. 3A and B), indicating a role for cellular components in the nucleation of arsenic sulfide nanomaterial. Absorbance at 375 nm (Abs_375_) was used to quantify the formation of arsenic sulfide, as reported in previous work (McFarlane *et al*., 2015). While there was an increase in Abs_375_ for the bottle containing arsenite and sulfide as shown in Fig. 3B, we did not see any arsenic sulfide precipitate after 72 h. The sample was centrifuged to collect any nanomaterials that may have formed, although no visible precipitate was collected and no structures were observed under SEM. The absorbance measurement likely detects smaller clusters of arsenite sulfide that may not be stable or observable under SEM. This result also suggests additional compounds and molecules provided by the cell might play a role in nucleation.

When arsenite and sulfide were added to anaerobic bottles with *E. coli* cells, we observed precipitation of arsenic sulfide material within hours (Fig. 3). To determine if live cells were required to initiate nanomaterial synthesis, we tested whether supernatant from *E. coli* culture would also lead to precipitation. *E. coli* cell culture was centrifuged and filtered using a 0.2 μm filter to obtain a cell‐free supernatant. The cell‐free supernatant also initiated nanomaterial synthesis (Fig.3C). The presence of cellular components resulted in a larger increase of absorbance at 375 nm, indicating production of arsenic sulfide. Unlike the arsenic sulfide spheres formed when arsenate As(V) and thiosulfate were added to cultures of *E. coli*, the addition of the reduced substrates to *E. coli* cultures formed nanofibres, as shown in Fig. 3D. In a control experiment shown in Table S3, we analysed whether the ratio of arsenite to sulfide influenced nanomaterial shape. We found that the ratios of reactants did not influence the shape, and only arsenic sulfide nanofibres were formed for all ratios. This confirms that active biogenic reduction process by a host organism influences the shape of the nanomaterial and not the ratio of concentrations of arsenite and sulfide available in the reaction.

We also tested the heat sensitivity of the cellular compounds involved in nanomaterial precipitation. Our results show that the cellular components involved in nucleation were not heat sensitive. Both autoclaved cell culture and autoclaved supernatant were capable of inducing precipitate formation. A summary of results and conditions tested with direct addition of arsenite and sulfide are summarized in Table S4. These results show that biomolecules secreted by the cells are necessary for the initiation of arsenic sulfide material precipitation. Unlike other metals, such as iron and cadmium that readily react with sulfide to form iron sulfide and cadmium sulfide complexes, under our experimental conditions arsenic sulfide requires a heat‐stable nucleating agent provided by cell. Similar experiments were conducted using the cultures of ANA‐3; live cells, autoclaved cells and supernatant were tested for their ability to precipitate arsenic sulfide nanomaterials in the presence of arsenite and sulfide, revealing trends similar to *E. coli* results (Table S4).

Additionally, we tested the ability of biological compounds found in media to nucleate arsenic sulfide nanomaterials. We tested if the organic molecules of biological origin found in a rich, complete media could induce precipitate formation, specifically LB, which contains yeast extracts, and minimal media with vitamins (see Table S5 for media components). These complex media also did not initiate arsenic sulfide precipitation. Results from abiotic experiments are summarized in Table 3. These results indicate that the compounds and molecules found that enable culture media to nucleate arsenic sulfide nanomaterials is somewhat specific to cultures of *E. coli* and ANA‐3. Previous reports have also found that microbes influence multiple aspects of nanomaterial formation (Singh *et al*., 2014; McFarlane *et al*., 2015; Hussain *et al*., 2016).

**Table 3:** Abiotic conditions tested for arsenic sulfide precipitation

Abiotic conditions	Reactants	Precipitation	Nanomaterial	Size (nm)
Buffer solution	Arsenite, sulfide	No	NA	NA
LB media	ARSENITE, sulfide	No	NA	NA
Minimal media with minerals and vitamins	Arsenite, sulfide	No	NA	NA

### Size dependence of arsenic sulfide nanofibres on synthesis conditions

Finally, we assessed whether biotic synthesis conditions influenced nanostructure dimensions. We compared nanofibres synthesized when arsenite, As(III) and sulfide were added to solutions of cells, cell‐free supernatant, autoclaved cells and autoclaved cell‐free supernatant for both *E. coli* and *Shewanella* sp. ANA‐3. SEM images of nanofibres from each condition were analysed using ImageJ (Abràmoff *et al*., 2004) to extract the diameter of each nanofibre.

**Figure 4:**
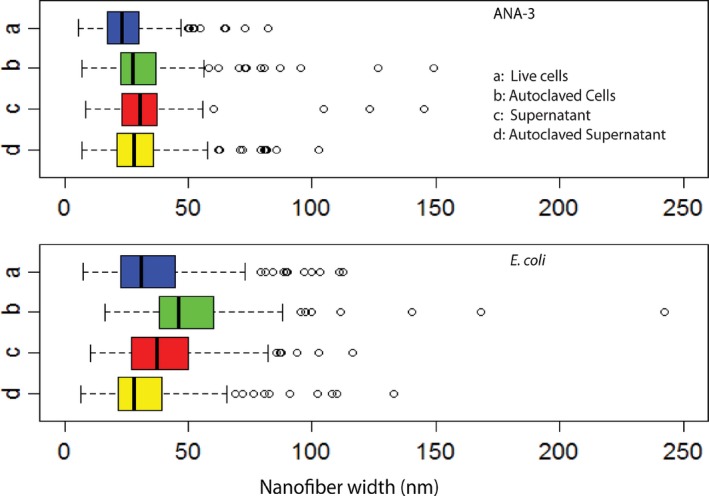
Size distribution of arsenic sulfide nanowires synthesized by the addition of arsenite and sulfide to cellular solutions derived from cultures of WT *Shewanella* sp. ANA‐3 (top) or engineered *E. coli* (bottom). Nanomaterials dimensions were calculated from SEM images. Size distribution in the box plot: the box represents the data in the 1st and 3rd quartile range; dark line in the middle of the box is the median size of the nanowire; the circles represent the outliers (outliers accounted for < 3% of measurements). A minimum of 450 fibres were analysed for each condition.

Solutions derived from *E. coli* cultures tended to form wider nanofibres than solutions derived from *Shewanella* sp. ANA‐3 cells. Although fibres from all conditions were similar in width, statistical analysis revealed significant shifts in fibre widths for some conditions (Table S6). In general, the size variation was larger for fibres produced in *E. coli* conditioned media (Fig. 4). These results demonstrate the choice of biogenic system for synthesis controls nanostructure properties including size.

## Discussion

We implemented synthetic genetic circuits in a heterologous host to synthesize biogenic nanostructures. As part of this work, we created two plasmids to recombinantly express arsenic reductases from *Shewanella* sp. ANA‐3 in *E. coli*. While *arsDABC* and *arrAB* pathways both enable *E. coli* to reduce arsenate to arsenite, *E. coli* was not able to respire arsenate for growth. Perhaps other respiratory biochemical pathways and cytochromes found in *Shewanella* are needed to utilize arsenate as the sole electron acceptor for growth. For example, a recent study demonstrated that both the MtrCAB cytochrome complex and the CymA cytochromes were needed for *E. coli* to respire and grow on iron(III) oxide (Jensen *et al*., 2016).

We used a co‐culture approach to synthesize arsenic sulfide nanomaterials: an *E. coli* strain expressing arsenate reductases from ANA‐3 and another *E. coli* strain expressing thiosulfate reductase from *Salmonella enterica* were used. Similar to our approach, reseachers used an engineered *E. coli* strain and *Sphingobium chlorphenolicum* for the bioremediation of hexachlorobenzene (Yan *et al*., 2015). A shift towards division of labour and the implementation of co‐cultures for bioprocessing has been explored in recent publications using synthetic microbial communities (Kim *et al*., 2011; Lindemann *et al*., 2016). The ability of bacteria to produce nanomaterials has been reported previously (Sweeney *et al*., 2004; da Costa *et al*., 2016; Choi *et al*., 2018), and here we explore how several biological parameters influenced the properties of the synthesized nanostructures.

For the formation of arsenic sulfide nanomaterial under ambient conditions, the presence of yet unknown biological components involved in nucleation were critical for material synthesis. In the absence of cells or cellular components, arsenic sulfide nanostructures did not precipitate out of solution. This points to an essential role for biological compounds in nucleating nanoscale materials. Nanomaterial nucleation is an active area of research, as understanding nucleation is critical to control the homogeneity of nanomaterials (Thanh *et al*., 2014). In some biological systems, the nucleating agent has been identified. Cadmium sulfide nanoparticles can nucleate on a cystathionine γ‐lyase enzyme (Dunleavy *et al*., 2016), and other known nucleation materials including enzymes, peptides and cells extracts have been reported for nanomaterial synthesis (Merzlyak and Lee, 2006; Spoerke and Voigt, 2007; Varma, 2012; Seker *et al*., 2017). For arsenic sulfide nanomaterial, the nucleating agent can be provided by either *E. coli* or ANA‐3 cells. We even found the supernatant of these cultures to be sufficient for nucleation of nanomaterial, although it is possible that supernatant contained cells fragments that lysed during preparation of the supernatant. The nucleating agent remained active after autoclaving the solution. The nucleation agent however was somewhat specific to these cultures. Media containing biological components such as digested proteins and vitamins did not precipitate material. These results demonstrate that microbes have multiple roles in nanostructure formation, not only processing starting materials, but also regulating the nucleation of materials.

Besides providing nucleation centres, the biological context of synthesis also influenced both the size and shape of arsenic sulfide nanomaterials. When substrates necessary for arsenic sulfide nanomaterials were processed by engineered *E. coli* spherical nanoparticles were formed, whereas all other conditions produced the nanofibres, similar to previous studies (Lee *et al*., 2007; McFarlane *et al*., 2015). Arsenic sulfide spheres have been observed in earlier work where the bacteria reduced arsenate to arsenite and in the presence of sulfide formed precipitates (Newman *et al*., 1997; Rodriguez‐Freire *et al*., 2016). In ANA‐3, even after the cells are dead, arsenic sulfide precipitation and yield continues to increase using dead cells and other biomolecules as a nucleation seed (Mcfarlane, 2015). Mechanisms that regulate nanoparticle shape were not determined. In other contexts, the host organism influenced material shape and size (Li *et al*., 2011; Dahoumane *et al*., 2017). For example, gold nanoparticles made by *Shewanella* were spherical (~12 nm) in shape (Suresh *et al*., 2011); however, *E. coli* produced triangles and hexagons (Du *et al*., 2007). However, in one study (Ramanathan *et al*., 2009), researchers demonstrated the shape of the silver nanomaterials synthesized depended on the growth kinetics of a silver resistant bacteria, *Morganella psychrotolerans*. Differences in the nanostructure dimensions were also dependent on the biological context of synthesis (Dahoumane *et al*., 2017), and here in our study, fibres derived from *E. coli* cultures were slightly wider and more heterogeneous in size. In a previous study, analyzing the formation of cadmium sulfide nanomaterials by *E. coli* only two of the four strains tested produced nanomaterials with photoluminescence (Sweeney *et al*., 2004), and in the same study stationary phase produced 20‐fold higher nanomaterials compared with log phase cells.

Together these results highlight the potential for control of nanoparticle properties by engineering the biological host system. Clearly the size, shape and yield of biogenic nanomaterials all depend on the choice of organism and culturing conditions. Arsenic sulfide nanomaterials, because of their semiconductor properties, have applications in the field of electronics and optoelectronics, i.e. photovoltaics and biosensors (Lee *et al*., 2007; McFarlane *et al*., 2015). Additionally, engineered strains expressing metal reductases could be used to synthesize various chlacogenides such as cadmium sulfide, zinc sulfide and cadmium selenide. Further research is needed to understand the mechanisms through which biological factors impact nanomaterial synthesis. Once characterized, pathways and molecules that control nanomaterials synthesis could be regulated to tune nanomaterial properties.

## Conclusions

Moving forward synthetic biological systems may be a valuable route of nanoparticle production. We demonstrated the ability to program a heterologous host to synthesize arsenic sulfide nanomaterials and have shown that the characteristics of the resulting materials depend on multiple biological factors. Manipulating the biological factors that impact nanomaterial characteristics using the ‘control knobs’ of synthetic biology has the potential to fine‐tune the properties of biogenic nanomaterials and may even enable the production of materials not possible through conventional methods.

## Experimental procedures

### Bacterial culture conditions

Bacterial strains were transferred from frozen stocks stored at −80°C and grown overnight under aerobic conditions in 5 ml of lysogeny broth. *Escherichia coli* (Δ*arsC*) strain JW3470‐1 was obtained from The Coli Genetic Stock Center, Yale University. The strain has arsenate reductase gene deleted by homologous recombination method. Deletion of *arsC* gene made *E. coli* more susceptible to arsenate toxicity (Diorio *et al*., 1995). Overexpressing *arsC* on a plasmid enabled cells to improve arsenate to arsenite reduction and hence improve the cell survival outcome (Diorio *et al*., 1995). The *arsC* mutant was used in these experiments to clarify that arsenate reduction was due to the ANA‐3 *arr* and *ars* pathways. Engineered *E. coli* strains containing plasmids to express the redox enzymes were grown at 37°C, and *Shewanella* sp. ANA‐3 cells were grown at 30°C in a shaker at 220 rpm. Upon reaching late log phase in LB, cells were transferred to anaerobic minimal media. Concentrations of vitamins, minerals and amino acids used in the anaerobic minimal media experiments are listed in Table S5. For *E. coli*, 15 mM glucose was added as a carbon source and for *Shewanella* sp. ANA‐3 15 mM lactate (ANA‐3) was used as a carbon source. The 30 mM fumarate was added as terminal electron acceptor for both *E. coli* and ANA‐3 cells. Cells grew under anaerobic conditions to reach an OD_600_ of 0.6–0.8.

All the stock solutions (sodium arsenate, sodium metaarsenite, sodium sulfide, sodium thiosulfate) were made anaerobic by flushing the solutions with nitrogen and sterilized by autoclaving them in anaerobic glass serum bottles. Appropriate antibiotics [spectinomycin (50 μg ml^−1^) and chloramphenicol (25 μg ml^−1^)] were added to all media to maintain plasmid selection.

### Arsenic sulfide nanomaterials synthesis

For experiments involving microbial reduction of substrates (arsenate and thiosulfate), we induced arsenate reductase genes in *E. coli* (OD_600_ of 0.6–0.8) using 2.5 mM IPTG and 0.5% arabinose and thiosulfate reductase gene using 2.5 mM IPTG. After 4 h of induction, we added 5 mM arsenate and/or 2.5 mM thiosulfate to the anaerobic serum bottles. These concentrations were chosen based on previous work (McFarlane *et al*., 2015).

In experiments to test the influence of cells and supernatant on the size and shape of the arsenic sulfide nanomaterial, late log phase cells of *E. coli* or ANA‐3 were centrifuged and washed three times with HEPES buffer to remove salts, and cells were then resuspended in buffer and added to sterile serum bottles. For testing cell culture supernatant, cells were filtered using 0.22 μm sterile filter and then added to sterile serum bottles, and oxygen was purged by flushing nitrogen through the system. In case of autoclaved cells or supernatants, cells or cell‐free supernatant and oxygen were purged by flushing nitrogen, and the bottles were autoclaved. Finally, 5 mM sodium metaarsenite and 10 mM sodium sulfide were added to the anaerobic bottles containing either cells or supernatant. Minimal media controls were tested for abiotic arsenic sulfide synthesis, and arsenic sulfide precipitate was not observed.

All reactions proceeded for 72 h, and samples were collected and imaged under scanning electron microscopy. Duplicate experiments were performed for each time point. To characterize relative abundance of arsenic sulfide nanomaterial, absorption at 375 nm was measured in standard cuvettes using a Nanodrop 2000C (ThermoFisher), as described previously (McFarlane *et al*., 2015).

### Nanomaterials shape and crystallinity analysis

Arsenic sulfide precipitate was separated from cells and media by centrifugation in 50 ml conical tubes. Precipitates were washed and resuspended, five times in DDI water to remove salts. After the washing process, the precipitate was resuspended in a 1 ml DDI water and deposited on a silicon wafer (Ted Pella) and air dried overnight. Samples were coated with gold in a sputter coater (Ted Pella), and scanning electron micrographs were obtained using JEOL 700 FE scanning electron microscopy, accessorized with energy‐dispersive X‐ray spectroscopy (EDX) for elemental analysis. Crystallinity of the nanomaterials was analysed by depositing the DI water washed nanomaterials on a glass slide and using a X‐ray diffraction (XRD) scans on a Rigaku Ultima IV diffractometer.

### Plasmid construction details

Plasmids expressing the arsenic reduction pathways from *Shewanella sp. *ANA‐3 were created using Gibson assembly. The *arsDABC* (GenBank: AY271410.1) and *arrAB* genes (GenBank: AY271310.1) were amplified from *Shewanella* sp. ANA‐3 and cloned into plasmids pZE (Reed *et al*., 2012) and pTSlb (Shis and Bennett, 2013). The pZE plasmid was modified for this experiment by replacing kanamycin resistance marker with spectinomycin resistance marker before adding *arsDABC* gene in place of GFP. The *arsDABC* expression was regulated by a IPTG inducible lac promoter, and arrAB was regulated by arabinose inducible pBAD promoter. For nanostructure synthesis, plasmids were transformed into *E. coli* strain JW3470‐1 ΔarsC (Baba *et al*., 2006) obtained from The Coli Genetic Stock Center, Yale University. This strain has a deletion of the *E. coli* arsenate reductase *arsC*. Note this gene is related to *arsC* gene from *Shewanella sp*. ANA‐3. ArsC is an integral protein involved in reduction of arsenate to arsenite; in the absence of this gene, arsenate reduction is inhibited in wild‐type *E. coli* and results in toxicity and cell death. Compare this *arrAB* genes, which are involved in arsenate respiration, and *E. coli* does not have the physiological abilities to respire arsenate and gain energy for its metabolic activities. A plasmid containing the thiosulfate reductase gene was obtained from Addgene, pSB74 (https://www.addgene.org/19591/). The gene was under an IPTG inducible promoter and had a carbenicillin resistance marker (Bang *et al*., 2000a,b).

### Arsenate reduction analysis

Concentrations of arsenite and arsenate species were measured using ICP‐MS (Exova, CA, USA). To prepare samples for ICP‐MS, *E. coli* cultures were incubated for 72 h with 5 mM arsenate As(V). Samples were centrifuged and filtered with 0.22 μm filter to remove cells. Arsenate reductase gene, if present, will reduce arsenate As(V) to arsenite As(III). If the cells do not possess arsenate reductase, or the genes were not active, arsenite production would be low or null similar to background levels.

### Thiosulfate reductase activity

*Escherichia coli* strain DH5alpha with pSB74 was tested for thiosulfate reduction. 1 mM thiosulfate was added to cultures after reaching at OD_600_ of 0.7. We measured the activity of thiosulfate reductase gene indirectly, the thiosulfate reductase enzyme converts thiosulfate to sulfide. There is a direct relationship between the gene activity of thiosulfate reductase and sulfide concentration. Cline assay was performed to measure the concentrations of sulfide using the procedure reported in (Bang *et al*., 2000a,b). Sulfide concentrations were measured using UV‐ Cuvette micro cuvettes in a NanoDrop 2000C spectrophotometer at 600 nm, and then concentrations were interpreted by comparing it with the calibrated reference solution of sodium sulfide. Thiosulfate reductase activity was also confirmed by adding 1 mM ferric chloride to the media (Bang *et al*., 2000a,b). This is a qualitative assay to confirm the production of sulfide by the bacteria; when expression of the thiosulfate reductase was induced, a black precipitate of iron sulfide was formed. Uninduced cultures formed less precipitate and no precipitate formed in cultures lacking the plasmid containing the thiosulfate reductase gene.

### Nanomaterial size analysis

Nanomaterial dimensions were determined from scanning electron microscopy (SEM) images. Objects in images were measured using ImageJ (Abràmoff *et al*., 2004). For images with nanofibres, a minimum of 150 fibre widths were measured per image. Three images were measured for each experimental condition.

## Conflicts of interest

None declared.

## Supporting information


**Fig. S1**. Maps of plasmids containing the *arsDACB* and *arrAB* genes involved in arsenic reduction cloned from *Shewanella sp*. ANA‐3.
**Fig. S2.** Schematic of arsenate reductase genes expressed in a heterologous host *E. coli* JWE470‐1 strain with deletion of native arsenate reductase arsC gene.
**Fig. S3.** No precipitate is formed within 72 h when sulfide and arsenate As(V) are added to the minimal media.
**Fig. S4.** Media bottles to show the *E. coli* cell culture turbidity in the absence and presence of arsenic sulfide nanomaterials.
**Fig. S5.** Arsenic sulfide material produced at 72 h by *E. coli* with either *arsDABC* or *arrAB* plasmid.
**Fig. S6.** Effect of nanoparticle formation on *E. coli* cell viability.
**Fig. S7.** EDX analysis of nanostructures synthesized by *E. coli* and ANA‐3.
**Fig. S8.** XRD pattern of arsenic sulfide nanomaterials synthesized by *E. coli* and ANA‐3.
**Table S1.** Primers used in this study.
**Table S2**. Amino acid sequence comparison of arsenate reductase system between *E. coli* and ANA‐3.
**Table S3.** Arsenic sulfide structures formed under different stoichiometric ratios of arsenite and sulfide directly added to bottles with *E. coli* cells.
**Table S4.** Influence of cells and cell‐free supernatant on arsenic sulfide nanomaterial dimensions.
**Table S5.** Concentrations of salts, amino acids and trace minerals mix used in the experiments to test the effects of abiotic media with minerals or amino acids on nucleation of arsenic sulfide nanomaterials.
**Table S6.** Student *t*‐test analysis to identify conditions that yielded statistically significant difference (numbers in bold) in arsenic sulfide nanofiber width (*P*<0.05) with *E. coli* and ANA‐3 as nucleation material.
