# Supplementary material for: Engineering bacteria for biogenic synthesis of chalcogenide nanomaterials
Source: Microb Biotechnol. 2018 Oct 17;12(1):161–72. doi: 10.1111/1751-7915.13320 (PMC6302716; doi:10.1111/1751-7915.13320)
Supplement: Supplementary file 1 — Fig. S1. Maps of plasmids containing the arsDACB and arrAB genes involved in arsenic reduction cloned from Shewanella sp. ANA‐3. Fig. S2. Schematic of arsenate reductase genes expressed in a heterologous host E. coli JWE470‐1 strain with deletion of native arsenate reductase arsC gene. Fig. S3. No precipitate is formed within 72 h when sulfide and arsenate As(V) are added to the minimal media. Fig. S4. Media bottles to show the E. coli cell culture turbidity in the absence and presence of arsenic sulfide nanomaterials. Fig. S5. Arsenic sulfide material produced at 72 h by E. coli with either arsDABC or arrAB plasmid. Fig. S6. Effect of nanoparticle formation on E. coli cell viability. Fig. S7. EDX analysis of nanostructures synthesized by E. coli and ANA‐3. Fig. S8. XRD pattern of arsenic sulfide nanomaterials synthesized by E. coli and ANA‐3. Table S1. Primers used in this study. Table S2. Amino acid sequence comparison of arsenate reductase system between E. coli and ANA‐3. Table S3. Arsenic sulfide structures formed under different stoichiometric ratios of arsenite and sulfide directly added to bottles with E. coli cells. Table S4. Influence of cells and cell‐free supernatant on arsenic sulfide nanomaterial dimensions. Table S5. Concentrations of salts, amino acids and trace minerals mix used in the experiments to test the effects of abiotic media with minerals or amino acids on nucleation of arsenic sulfide nanomaterials. Table S6. Student t‐test analysis to identify conditions that yielded statistically significant difference (numbers in bold) in arsenic sulfide nanofiber width (P<0.05) with E. coli and ANA‐3 as nucleation material. [file MBT2-12-161-s001.docx]

Supplementary Information for

**Engineering bacteria for biogenic synthesis of chalcogenide nanomaterials**

Prithiviraj Chellamuthu, Frances Tran, Kalinga Pavan T. Silva, Marko S. Chavez, Mohamed Y. El-Naggar, James Q. Boedicker


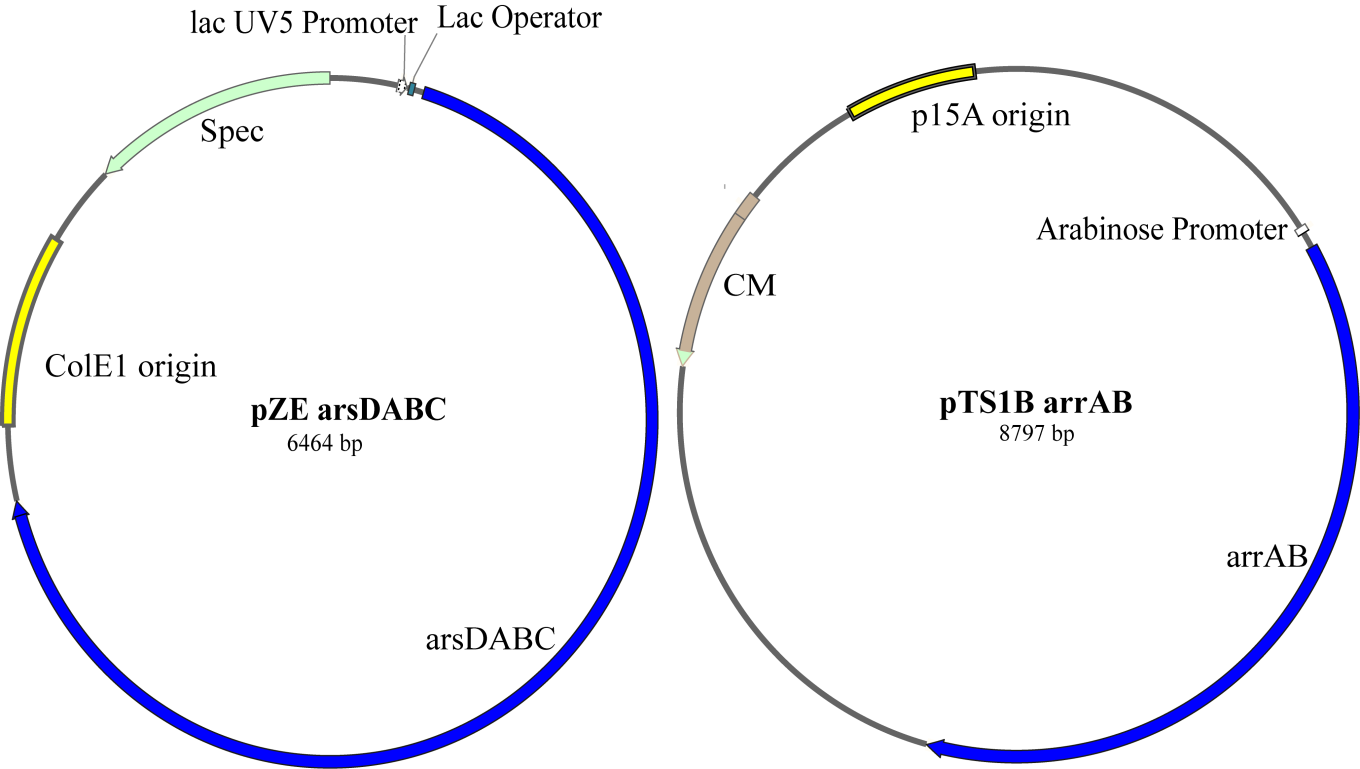


Figure S1: Maps of plasmids containing the *arsDACB* and *arrAB* genes involved in arsenic reduction cloned from *Shewanella sp.* ANA-3. These plasmids enabled host *E. coli* cells to reduce As(V) to As(III).

**Table S1: Primers used in this study**

| Primer | Sequence | Comments |
| --- | --- | --- |
| pBAD-arrAB_F | AGATCAGGGCCAGGTATGAAGAAAGAGAATCAAGTCAAC | Amplify ArrAB gene for Gibson Assembly Integration (F) |
| pBAD-arrAB_R | GCAGGTCGACTCTAGATTAATAAGCGGTTTTAACACC | Amplify ArrAB gene for Gibson Assembly Integration (R) |
| pBAD-F | TCTAGAGTCGACCTGCAGGC | Amplify pBAD plasmid backbone for arrAB integration (F) |
| pBAD-R | ACCTGGCCCTGATCTTTATATATAGG | Amplify pBAD plasmid backbone for arrAB integration (R) |
| pZE-arsDABC_F | AAAGAGGAGAAAGGTACCGCATGACTCATTTTTCGATATTCG | Amplify ArsDABC gene for Gibson Assembly Integration (F) |
| pZE-arsDABC_R | CTAGACTCAGCTAATTAAGCGTTGCTTAAGTTTTAGTATGG | Amplify ArsDABC gene for Gibson Assembly Integration (R) |
| pZE-F | GCTTAATTAGCTGAGTCTAGAGG | Amplify pZE plasmid backbone for arsDABC integration (F) |
| pZE-R | GCGGTACCTTTCTCCTCTTTAATG | Amplify pZE plasmid backbone for arsDABC integration (F) |

**
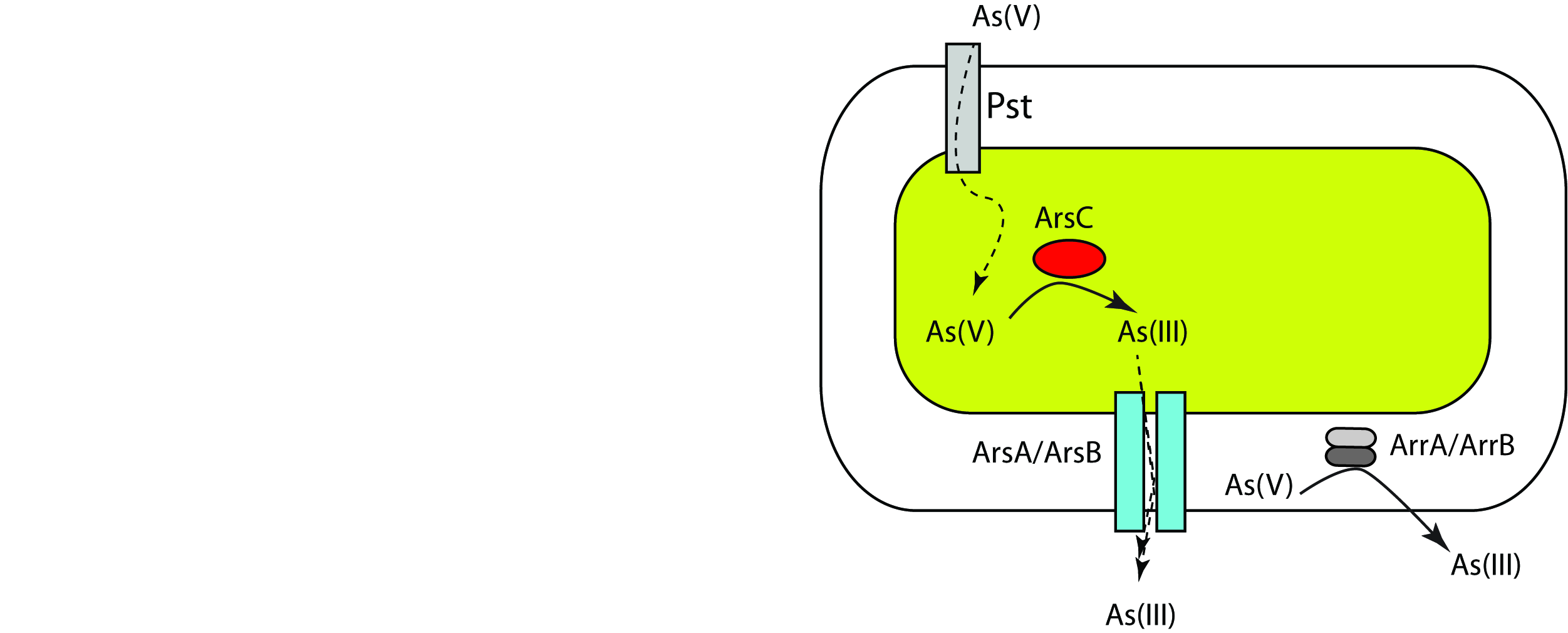
**

**Figure S2: Schematic of arsenate reductase genes expressed in a heterologous host *E. coli* JWE470-1 strain with deletion of native arsenate reductase *arsC* gene. *arsDAB*C is a detoxification pathway, and *arrAB* is an arsenate reductase respiratory pathway, both genes were cloned from *Shewanella sp.* ANA-3. In *E. coli*, arsenate gets transported into the cytoplasm via phosphate transporter Pst; in the cytoplasm, ArsC reduces arsenate to arsenite; ArsA and ArsB extrude the reduced arsenite from the inside of the cell to the outside to reduce toxicity. ArrA and ArrB are heterodimeric proteins involved in arsenate reduction in the periplasmic space and the reduced arsenite is transported to the outside of the cells.**

**Table S2:**

| Gene (*E. coli*/ ANA-3) | Amino acid length | Amino acid sequence Identity | Protein function |
| --- | --- | --- | --- |
| *arsR/arsD* | 117/120 | 25% | Arsenate concentration dependent regulation of the arsenate detoxification operon. |
| *arsB/arsB* | 429/426 | 85% | Extrudes arenite from the cytoplasm, where arsenate is reduced to arsenite, to the outside of the cell to minimize toxicity. |
| *arsC/arsC* | 141/158 | 73% | Reduces arsenate to arsenite. |

**Abiotic arsenate reduction by sulfide**

Chemical reduction of arsenate by sulfide has been reported earlier, but the rate of reduction and formation of arsenic sulfide precipitate takes extended period of time. We tested 5 mM arsenate reduction with 10 mM sulfide, but there was no visible yellow precipitate in the time period tested (72 hours).

**Figure S3: No precipitate is formed within 72 hours when sulfide and arsenate As(V) are added to the minimal media. Left most bottle (control media, no arsenate, no sulfide, no cells), center and right most bottle are duplicates with arsenate and sulfide without cells.**


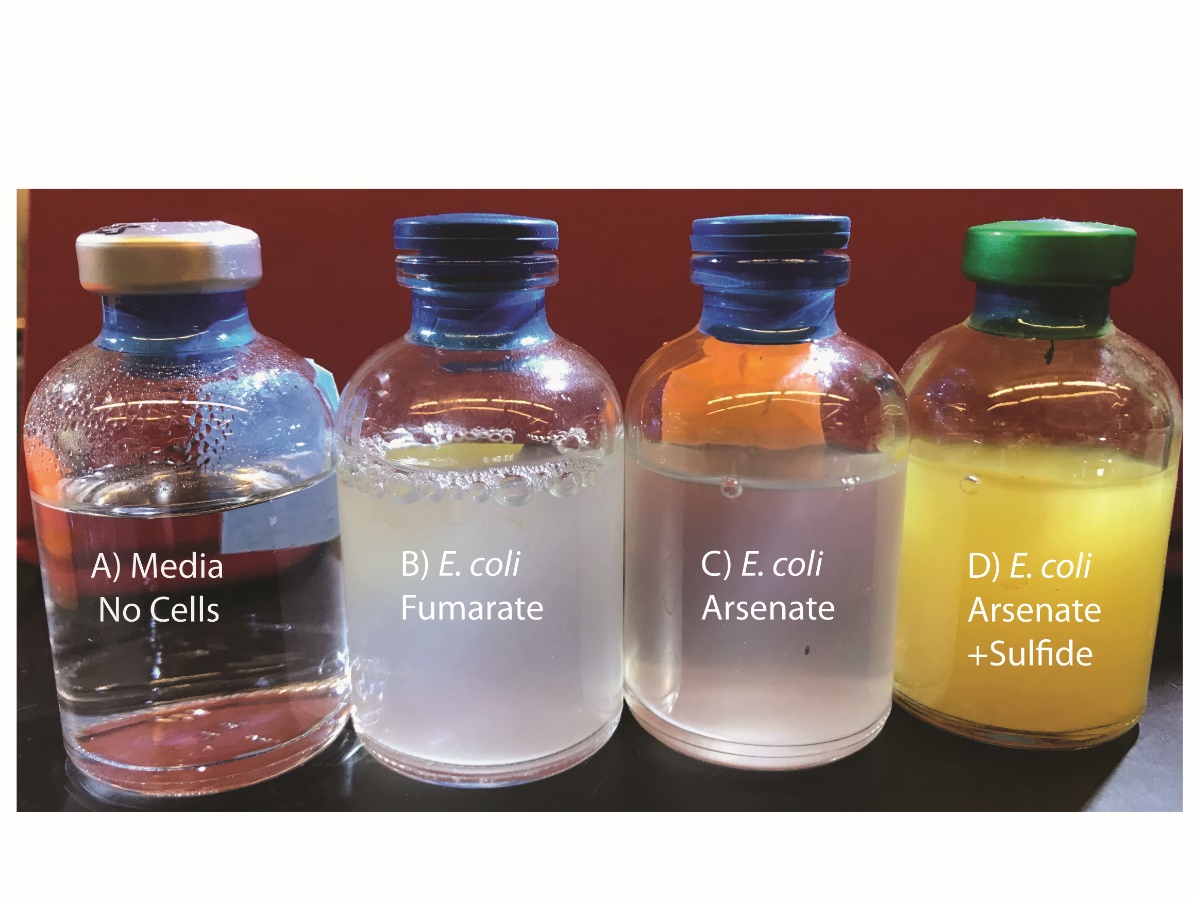


**Figure S4: Media bottles to show the *E. coli* cell culture turbidity in the absence and presence of arsenic sulfide nanomaterials. A) bottle with no cells and no arsenic sulfide nanomaterial; B) *E. coli* cells with fumarate, a non-toxic electron acceptor; C) *E. coli* cells with arsenate but not sulfide; D) E. coli cells with arsenate and sulfide that formed arsenic sulfide nanomaterials.**


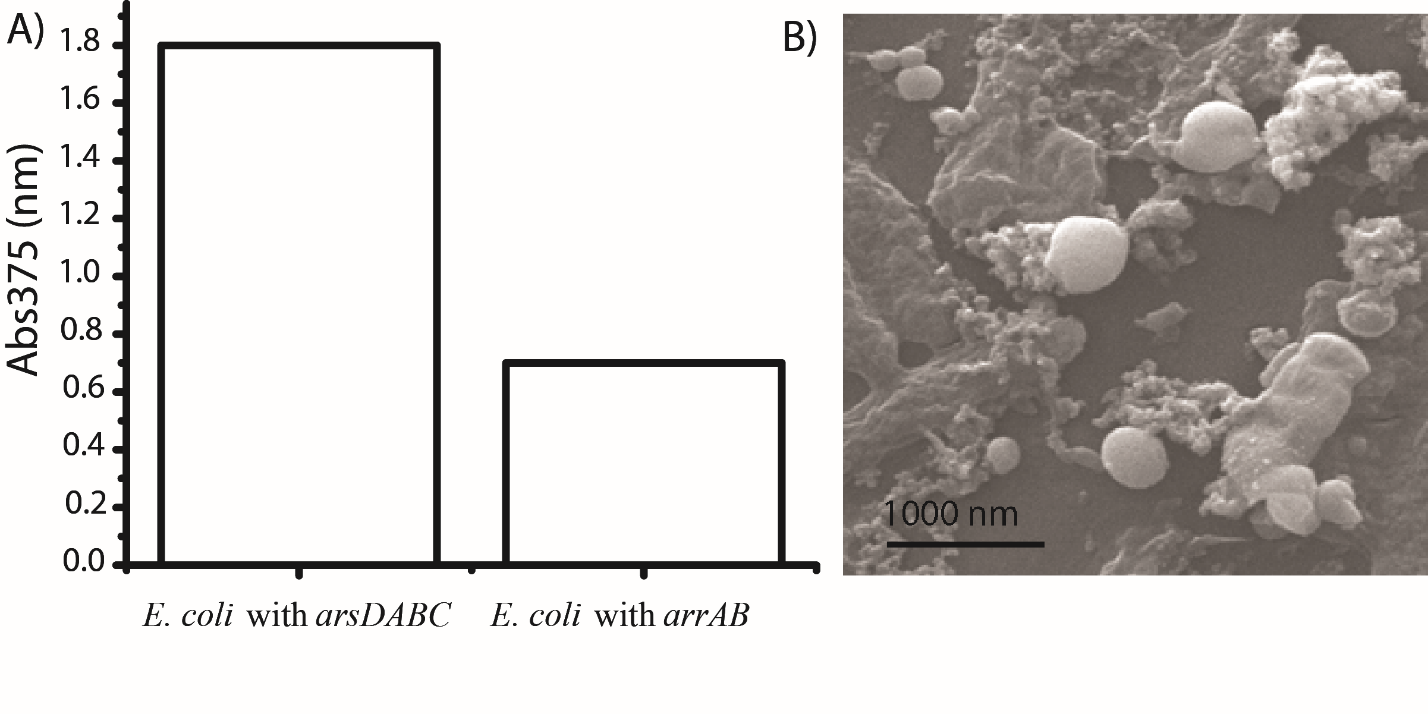
**Figure S5: Arsenic sulfide material produced at 72 hours by *E. coli* with either *arsDABC* or *arrAB* plasmid. A) Arsenic sulfide nanomaterial production measured by Abs_375_ nm by *E. coli* cells with *arrAB* plasmid and *arsDABC* plasmid alone. B) Scanning electron micrograph of nanospheres synthesized by *E. coli* cells with *arsDABC* plasmid. Cells with *arrAB* also produced nanospheres.**

**
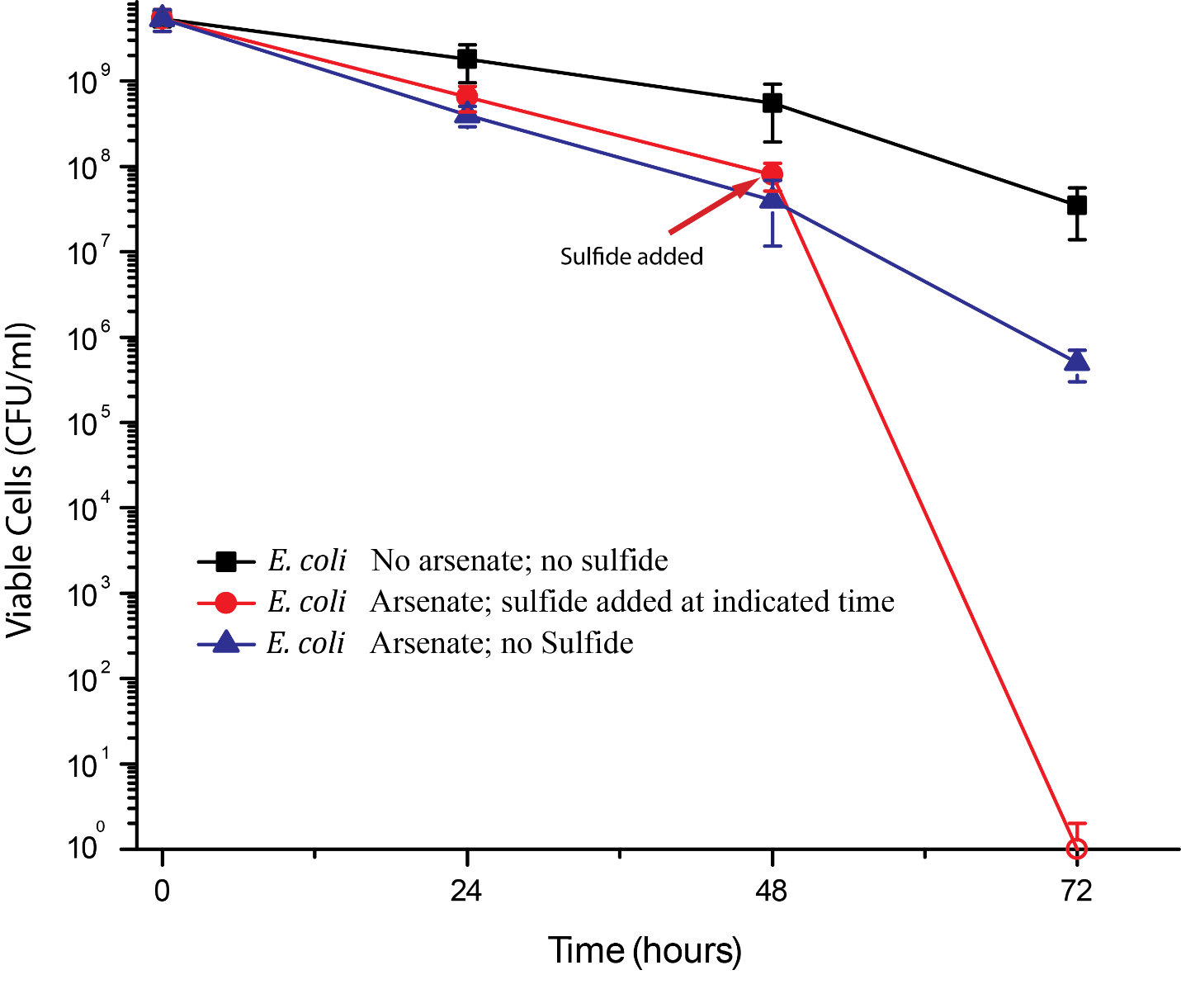
**

**Figure S6: Effect of nanoparticle formation on *E. coli* cell viability. Cell viability was monitored by plating cells on LB agar and counting colonies to identify the number of viable cells. At 48 hours, when sulfide was added to the culture, arsenic sulfide nanomaterial formation initiated as confirmed by the formation of yellow precipitate. The cell viability dropped to less than 10 cells per ml within a 24 hour period. The open circle at 72 hours indicates cell density less than 1 CFU/mL. Cells with arsenate and no sulfide had a drop in viable cells compared to cells without arsenate.**


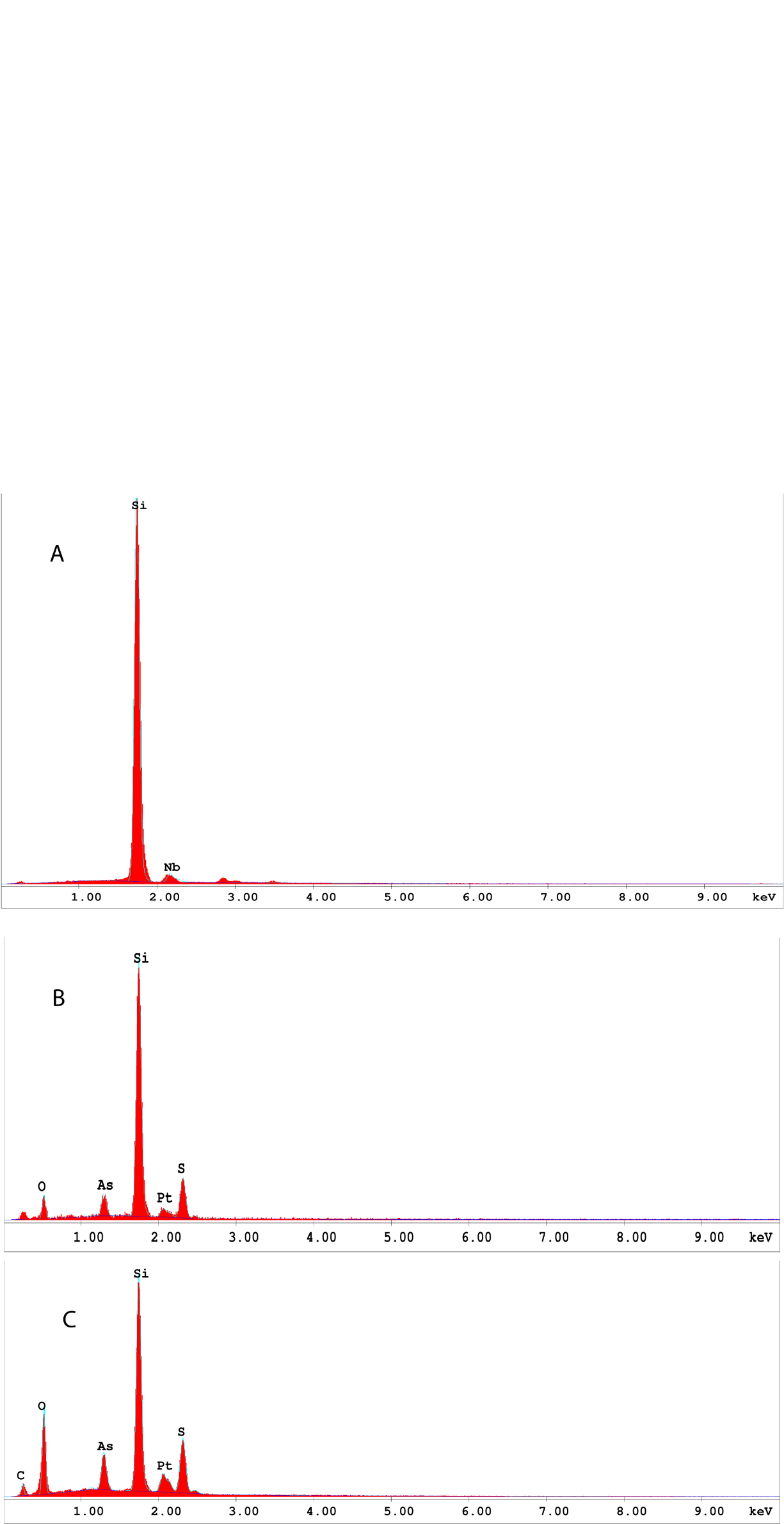


**Figure S7: EDX analysis of nanostructures synthesized by *E. coli* and ANA-3. A) Control silicon substrate shows no arsenic or sulfur peaks. Arsenic and sulfide peaks were observed in materials made by engineered *E. coli* (B) and wildtype *Shewanella sp.* ANA-3 (C).**

**Figure S8: XRD pattern of arsenic sulfide nanomaterials synthesized by *E. coli* and ANA-3. The material synthesized by both organisms contained multiple crystalline phases with several overlapping peaks associated with crystalline structures assigned to realgar, orpiment, and alacranite.**

**Table S3: Arsenic sulfide structures formed under different stoichiometric ratios of arsenite and sulfide directly added to bottles with *E. coli* cells.**

| Arsenite (mM) | Sulfide (mM) | As:S ratio | Structures |
| --- | --- | --- | --- |
| 5 | 10 | 1:2 | Nanofibers |
| 5 | 5 | 1:1 | Nanofibers |
| 5 | .5 | 10:1 | Nanofibers |

Table S4: Influence of cells and cell-free supernatant on arsenic sulfide nanomaterial dimensions

| Biotic Conditions | Reactants | Size (nm) |
| --- | --- | --- |
| Live ANA-3 cells | arsenite, sulfide | 31+/-15 |
| Live *E. coli* cells | arsenite, sulfide | 32+/-18 |
| Autoclaved ANA-3 cells | arsenite, sulfide | 32+/-17 |
| Autoclaved *E. coli* cells | arsenite, sulfide | 52+/-26 |
| Autoclaved ANA-3 supernatant | arsenite, sulfide | 24+/-11 |
| Autoclaved *E. coli* supernatant | arsenite, sulfide | 36 +/-20 |
| ANA-3 supernatant | arsenite, sulfide | 32+/-15 |
| *E. coli* supernatant | arsenite, sulfide | 40+/-18 |

**Table S5: Concentrations of salts, amino acids and trace minerals mix used in the experiments to test the effects of abiotic media with minerals or amino acids on nucleation of arsenic sulfide nanomaterials**

| Salts | Concentration (mM) |
| --- | --- |
| HEPES buffer | 30 |
| Ammonium chloride | 28 |
| Potassium chloride | 1.34 |
| Sodium phosphate monobasic | 4.35 |
| Sodium hydroxide | 20 |
| Sodium DL-lactate | 20 |

| Amino Acid | Concentration (mM) |
| --- | --- |
| DL-serine | 1.90 x 10^-1^ |
| L-Arginine | 1.15 x 10^-1^ |
| L-Glutamic Acid | 1.36 x 10^-1^ |

| Minerals and vitamins | Concentration (mM) |
| --- | --- |
| Aluminum Potassium Disulfate Dodecahydrate | 2.11 x 10^-4^ |
| Biotin | 8.19 x 10^-5^ |
| Boric Acid | 1.62 x 10^-3^ |
| Calcium Chloride Dihydrate | 6.80 x 10^-3^ |
| Cobalt Chloride Hexahydrate | 4.20 x 10^-3^ |
| Cupric Sulfate Pentahydrate | 4.01 x 10^-4^ |
| d-Pantothenic Acid, Hemicalcium Salt | 2.10 x 10^-4^ |
| Ferrous Sulfate Heptahydrate | 3.60 x 10^-3^ |
| Folic Acid | 4.53 x 10^-5^ |
| Magnesium Sulfate Heptahydrate | 1.22 x 10^-1^ |
| Manganese Sulfate Monohydrate | 2.96 x 10^-2^ |
| Nicotinic Acid | 4.06 x 10^-4^ |
| Nitrilotriacetic Acid | 7.85 x 10^-2^ |
| p-Aminobenzoic Acid | 3.65 x 10^-4^ |
| Pyridoxine HCl | 4.86 x 10^-4^ |
| Riboflavin | 1.33 x 10^-4^ |
| Sodium Molybdate Dihydrate | 1.03 x 10^-3^ |
| SodiumTungstate | 7.58 x 10^-4^ |
| Thiamine HCl monohydrate | 1.41 x 10^-4^ |
| Thioctic Acid | 2.42 x 10^-4^ |
| Vitamin B12 | 7.38 x 10^-7^ |
| Zinc Chloride | 9.54 x 10^-3^ |
| Nickel Chloride Hexahydrate | 1.01 x 10^-3^ |

**Table S6: Student t-test analysis to identify conditions that yielded statistically significant difference** **(numbers in bold) in arsenic sulfide nanofiber width (p<0.05) with *E. coli* and ANA-3 as nucleation material.**

| *E. coli* | | | | |
| --- | --- | --- | --- | --- |
| Condition | Cells | Supernatant | Autoclaved cells | Autoclaved supernatant |
| Cells | 1 | **2.3 x 10^-5^** | **1.4 x 10^-14^** | 0.04 |
| Supernatant | **-** | 1 | 1**.7 x 10 ^-6^** | 0.05 |
| Autoclaved cells | **-** | **-** | 1 | **2.6*10 ^-9^** |
| Autoclaved supernatant | - | - | **-** | 1 |

| *Shewanella* sp. ANA-3 | | | | |
| --- | --- | --- | --- | --- |
| Condition | Cells | Supernatant | Autoclaved cells | Autoclaved supernatant |
| Cells | 1 | 0.47 | 0.57 | **1.5 *10^-6^** |
| Supernatant | - | 1 | 0.91 | **6.2 *10 ^-8^** |
| Autoclaved cells | - | - | 1 | **7.6 *10 ^-7^** |
| Autoclaved supernatant | **-** | **-** | **-** | 1 |
